# Supplementary material for: Inhomogeneous Kondo-lattice in geometrically frustrated Pr2Ir2O7
Source: Nat Commun. 2021 Mar 2;12:1377. doi: 10.1038/s41467-021-21698-z (PMC7925525; doi:10.1038/s41467-021-21698-z)
Supplement: Supplementary file 1 — Supplementary Information [file 41467_2021_21698_MOESM1_ESM.pdf]

Supplementary Material for "**Inhomogeneous Kondo lattice in geometrically frustrated $Pr_2Ir_2O_7$**"

Mariam Kavai[1]†, Joel Friedman[1]†, Kyle Sherman[1]†, Mingda Gong[1], Ioannis Giannakis[1], Samad Hajinazar[1], Haoyu Hu[2], Sarah E. Grefe[2], Justin Leshen[1], Qiu Yang[3], Satoru Nakatsuji[3-5], Aleksey N. Kolmogorov[1], Qimiao Si[2], Michael Lawler[1], and Pegor Aynajian[1]*

[1]Department of Physics, Applied Physics and Astronomy, Binghamton University, Binghamton, New York 13902, USA
[2]Department of Physics and Astronomy, Rice Center for Quantum Materials, Rice University, Houston, Texas, 77005, USA
[3]Institute for Solid State Physics, University of Tokyo, Kashiwa, Chiba 277-8581, Japan
[4] Department of Physics, University of Tokyo, Bunkyo-ku, Tokyo 113-0033, Japan
[5]Trans-scale Quantum Science Institute, University of Tokyo, Bunkyo-ku, Tokyo 113-0033, Japan

* To whom correspondence should be addressed: aynajian@binghamton.edu
†Equal contribution

Supplementary Note 1

Supplementary Fig. 1 represents three topographs (size: 65nm, 32 nm, and 16 nm) measured on the cleaved Pr227, along with their corresponding Fourier transforms. All images show the atomic resolution and the Kagome structure. Each Fourier transform shows sharp Bragg peaks at the corresponding $2\pi/a$, which reflects a strongly periodic signal in the topograph.

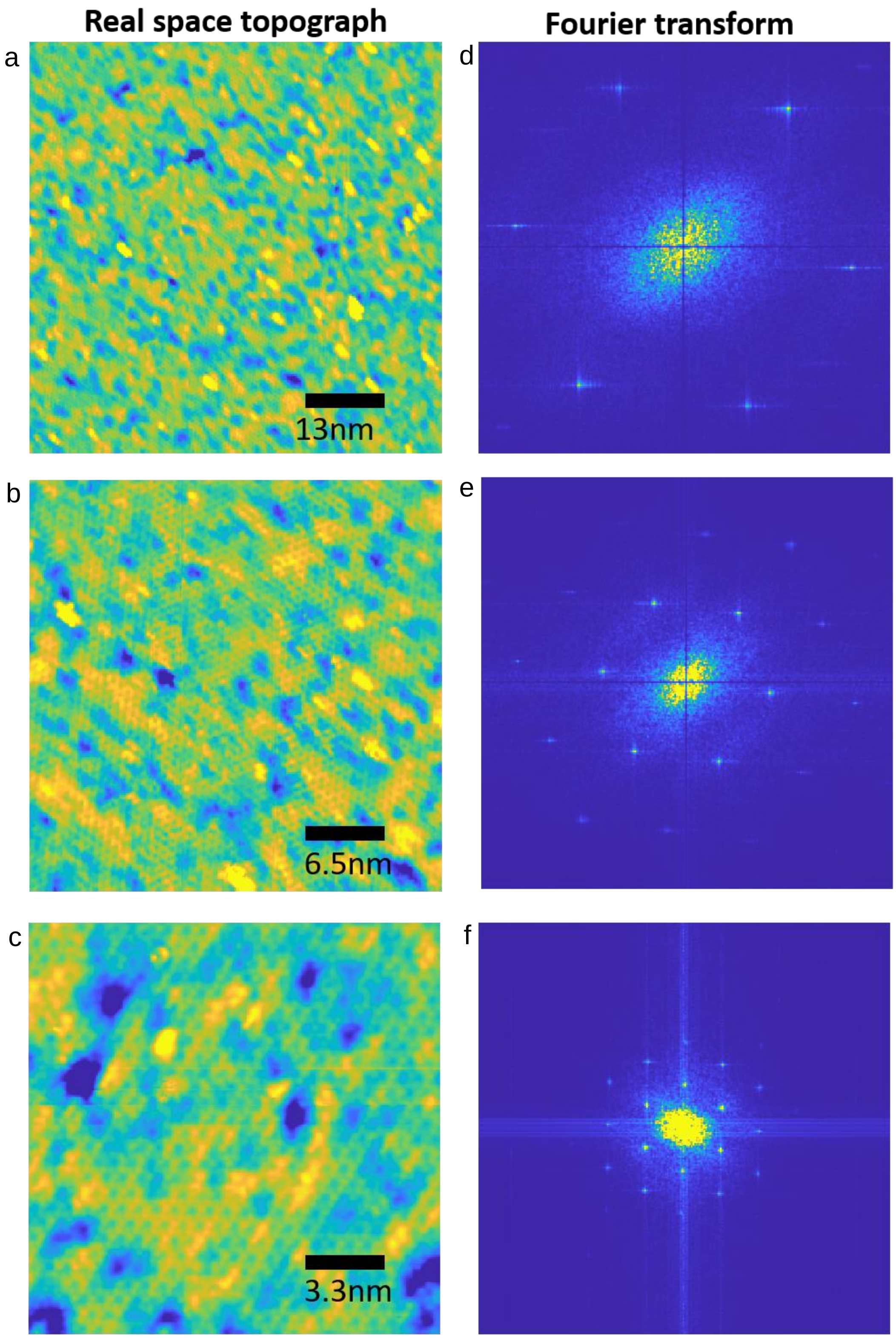

Supplementary Figure 1. Atomically resolved topographs (a-c) and their corresponding Fourier transforms (d-f) measured at T = 33 K. All topographs are obtained with a 256x256 pixels. As the size of the topographs decrease, the 6-fold Bragg peaks in their corresponding Fourier transform move closer to q=0 and the higher order Bragg peaks become visible. The large central peak at q=0 corresponds to long wavelength inhomogeneity.

Supplementary Note 2

Overview of DFT studies on $Pr_2Ir_2O_7$

It has been shown experimentally that $Pr_2Ir_2O_7$ in its ground state (GS) is a metallic compound with no long-range magnetic order[1–7]. Only two computational studies have been conducted at the density functional theory (DFT) level on characterizing the $Pr_2Ir_2O_7$ GS[8,9]. Simulations with the the local density approximation (LDA)+U approach have shown that a paramagnetic semi-metallic state favored at $U_{eff} = 0$ becomes antiferromagnetic (AFM) semi-metallic at U_{eff} values of ~1.3 eV and eventually AFM insulating at U_{eff} ~1.5-1.6 eV[8], in disagreement with the experimental observations. More recently, Zhang *et. al.*[9] argued that the LDA+U approach is not a proper treatment for $Pr_2Ir_2O_7$ due to the intrinsic tendency of the +U correction to favor magnetic insulating states. The study relied on the LDA+DMFT (dynamical mean field theory)[10] approach and successfully reproduced the experimentally observed properties of the $Pr_2Ir_2O_7$ GS.

The primary focus of our DFT calculations was to (i) establish energetically favorable surface terminations in cleaved $Pr_2Ir_2O_7$ samples at zero temperature and (ii) simulate STM images of possible terminations to help interpret experimental observations. The significant computational cost of the LDA+DMFT treatment[11] makes it an impractical approach for the analysis of complex surfaces. In order to identify a suitable DFT flavor for this study, our starting point was the examination of $Pr_2Ir_2O_7$ GS properties within three commonly used DFT[12,13] approaches: the generalized gradient approximation (GGA) with the Perdew–Burke–Ernzerhof (PBE) functional[14], PBE+U[15,16], and Heyd–Scuseria–Ernzerhof (HSE) hybrid functionals[17].

Tests of DFT approximations

Our benchmarking included (i) a full optimization of the $Pr_2Ir_2O_7$ bulk structure with the PBE and PBE+U approaches; and (ii) comparison of the magnetic order in the PBE, PBE+U, and HSE treatments against the experimental observations. For the PBE (+U) treatments, we also examined the effect of the spin-orbit coupling (SOC) and found it to be insignificant for the relevant structural, magnetic, and stability properties of $Pr_2Ir_2O_7$.

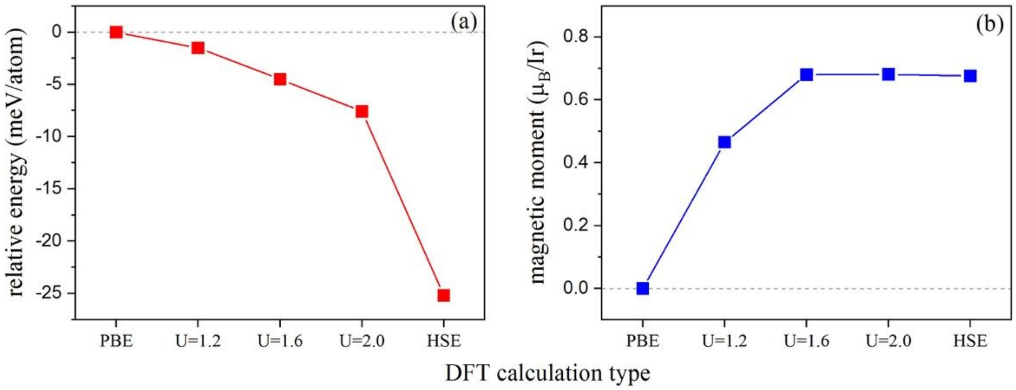

Supplementary Figure 2. a Relative energy of the ferromagnetic vs. non-magnetic bulk structures within various DFT treatments, and **b** average magnetic moment per Ir atom in the ferromagnetic bulk structure optimized with different DFT approaches.

All DFT calculations were based on the PBE parametrization of the generalized gradient approximation (GGA)[18] and the projector augmented wave (PAW) potentials[19] as implemented in VASP[20,21]. A high energy cut-off of 500 eV and a dense *k*-point sampling of the Brillouin zone within the Monkhorst-Pack approach[22] were employed to ensure the numerical convergence of the calculations (5×5×5 for the benchmarking tests and 6×6×6 and 6×6×1 for the final calculations of bulk and slab structures, respectively). In the PBE+U treatment, we optimized the bulk GS with three values of U_{eff}: 1.2, 1.6, and 2.0 eV. For the HSE hybrid functional calculations, we used the HSE06[23] range-separated functional with 25% of short-range exact exchange interaction mixed, and chose to not downsample the Brillouin zone in the calculation of the exact non-local exchange term to achieve higher accuracy. As a common practice, the computationally demanding HSE calculations were performed for the structure fully optimized at the PBE level.

Supplementary Fig. 2a shows the energy difference between the $Pr_2Ir_2O_7$ bulk structure configurations with non-magnetic and collinear ferromagnetic (FM) initializations of atomic moments. Among the considered DFT flavors, only the PBE treatment correctly did not stabilize an (A)FM ordering, while the PBE+U and HSE approximations produced lower-energy states with significant FM moments on Ir atoms (Supplementary Fig. 2b). In the SOC non-collinear calculations, we extended the set of magnetic moment initializations with all-in-all-out and 2-in-2-out AFM configurations for Pr atoms in combination with various initial values for the Ir and O atoms and observed the same pattern: a non-magnetic state with PBE and non-zero magnetic moments on Ir atoms in PBE+U for all considered U>0 eV values. We determined that the fully relativistic treatment had little effect on the structural parameters of the PBE-relaxed structure: inclusion of the SOC produced forces below 0.03 eV/Å and changed the lattice constant by less than 0.15%. Given this minor impact of the SOC on the properties relevant for the current study and the high computational cost of the fully relativistic treatment, we chose to rely on the PBE calculations in the simulation of $Pr_2Ir_2O_7$ surfaces.

Surface structures and exfoliation energies

Full optimization of the cF88-$Pr_2Ir_2O_7$ structure with space group #227 at the PBE level resulted in lattice parameter *a* = 10.512 Å and Wyckoff positions 48*f* (0.4190,1/8,1/8) and 8*a* (1/8,1/8,1/8) for O, 16*d* (1/2,1/2,1/2) for Ir, and 16*c* (0,0,0) for Pr atoms. This structure was used to construct the slabs for comparing the surface stabilities.

As illustrated in Supplementary Fig. 3a, the bulk $Pr_2Ir_2O_7$ GS structure in the (111) direction consists of alternating Pr- and Ir-rich layers (which will be referred to as A and B layers). Each O is bonded to 3 metal atoms within the layer and 1 metal atom in a neighboring layer, while the metal coordinations for bulk are PrO_8 and IrO_6. In isolated layers, the coordinations are ($3PrO_6$ & $1IrO_6$) for A and ($1PrO_6$ & $3IrO_4$) for B, so that one can assign ($3xO_6$+$1xO_6$)/3= 8 O atoms to A layers and ($1xO_6$+$3xO_4$)/3= 6 O atoms to B. Three relevant slab types can be constructed with the following surface terminations: $[AB]_n$, $[AB]_nA$, and $[BA]_nB$, where *n* is the number of full formula units in the slab. The $[AB]_n$ slabs are non-symmetric but stoichiometric (Supplementary Fig. 3a), while $[AB]_nA$ and $[BA]_nB$ slabs are symmetric but non-stoichiometric (Supplementary Fig. 3b,c).

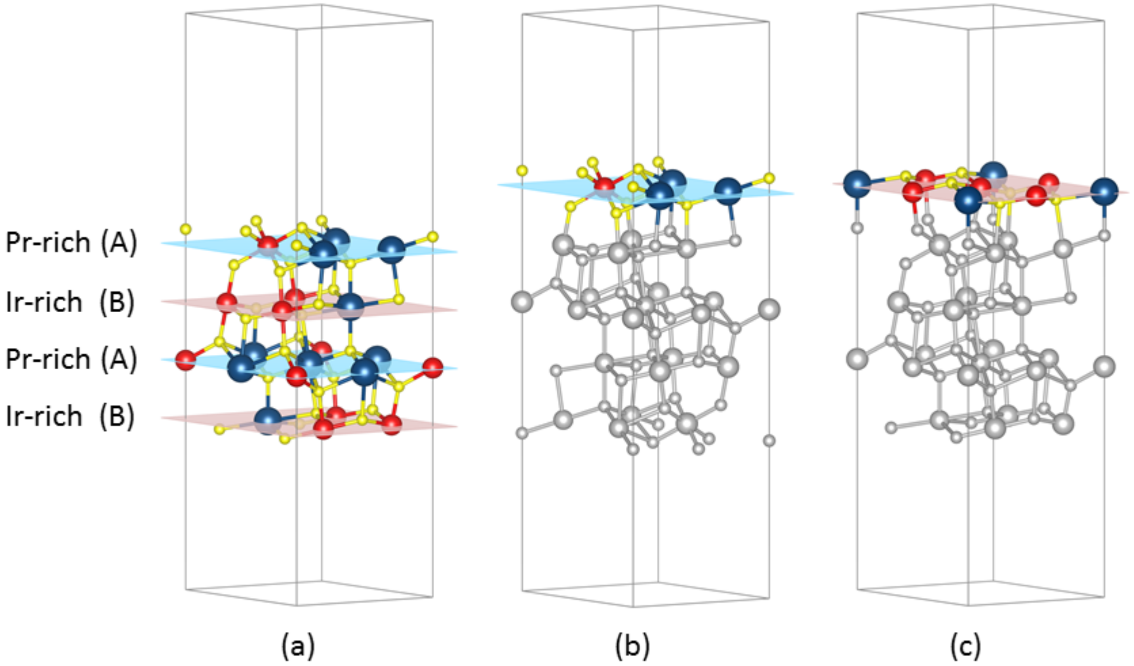

Supplementary Figure 3. $Pr_2Ir_2O_7$ (111) slabs are formed from Pr- and Ir-rich layers (A and B layers, respectively). **a** A non-symmetric stoichiometric $[AB]_2$ slab contains two formula units of the bulk structure with O, Ir, and Pr atoms shown in yellow, red, and blue, respectively. **b** A symmetric non-stoichiometric $[AB]_2A$ slab includes two formula units of bulk (shown with grey color) and an A layer. **c** A symmetric non-stoichiometric $[BA]_2B$ slab with two bulk units (shown with grey color) and an extra B layer.

The typical procedure in the calculation of surface energy is to construct slabs of the same termination and then calculate the surface energy[24] as

$$E_{surf} = \frac{1}{2A}(E_{slab} - nE_{bulk})$$

in which A is the surface area, E_{slab} is the total DFT energy of the optimized slab, *n* is the number of bulk formula units in the slab, and E_{bulk} is the DFT energy of the bulk structure per formula unit. However, since the $[AB]_nA$ and $[BA]_nB$ slabs are non-stoichiometric, this equation cannot be used to determine the surface energy of the A and B terminations separately. Supplementary Fig. 4b shows the convergence of the combined (A+B) surface energy as a function of the number of [AB] units in the stoichiometric slabs. The results indicate that converged results for surface stability can be obtained for slabs with only two [AB] units (44 atoms). The value of 132 meV/ Å^2 (6.3 eV/u.c. of (111) slab) can be attributed primarily to the break of 3 Ir-O and 4 Pr-O bonds per u.c. of the slab. Supplementary Fig. 4(b) also illustrates that both A and B surfaces undergo similar degrees of structural rearrangement, as the energy differences between relaxed and unrelaxed terminations are close to 10 meV/ Å^2 for each side.

The stability of the individual A and B terminations can also be probed via the exfoliation of single layers. For the *X* = A and B, the exfoliation energy[24] is defined as:

$$E_{exf.} = \frac{1}{A}\left(E^{X} + E^{[\mathrm{AB}]n} - E^{[\mathrm{AB}]nX}\right)$$

in which E^X is the energy of a single layer *X* and all energies are obtained after full optimization of the atomic positions in the slab. For each PBE calculation, we used the above-mentioned settings while the

generated slab was separated with a 15 Å vacuum in the non-periodic direction to isolate the periodic boundary condition's effects in the DFT run. In geometry optimizations, all atoms were allowed to move while the lattice parameters were kept fixed. Moreover, to ensure that electric dipole effects does not affect our calculations, we ran a set of optimization with dipole correction[25] included and observed no difference in optimized geometries and the energy shift was ~0.5 meV/atom.

Supplementary Fig. 4a shows the exfoliation energy for $[AB]_nA$ and $[BA]_nB$ for n up to 4. According to these calculations, the exfoliation energies for A and B layers are 158 and 62 meV/Å^2, respectively. Note that their sum of 220 meV/Å^2 is lower than the doubled value of the surface energy of 2×132 = 264 meV/Å^2, so it is more energetically favorable to lift two individual A and B layers than to create two new cuts through the middle of the sample.

It is important to note that transfer of top-most O atoms between A- and B-terminated surfaces during sample cleavage is expected to be uncommon because of unfavorable energetics. Our PBE calculations for stoichiometric $[AB]_3$ slabs revealed that changing the natural 4:3 ratio of O atoms covering the respective Pr- and Ir-rich surfaces to 3:4 (5:2) raises the total energy by 1.2 (3.6) eV. The PBE+SOC calculations produce similar 1.2 (3.8) eV penalties. Therefore, cleaved (111) surfaces are expected to retain the natural number of O atoms.

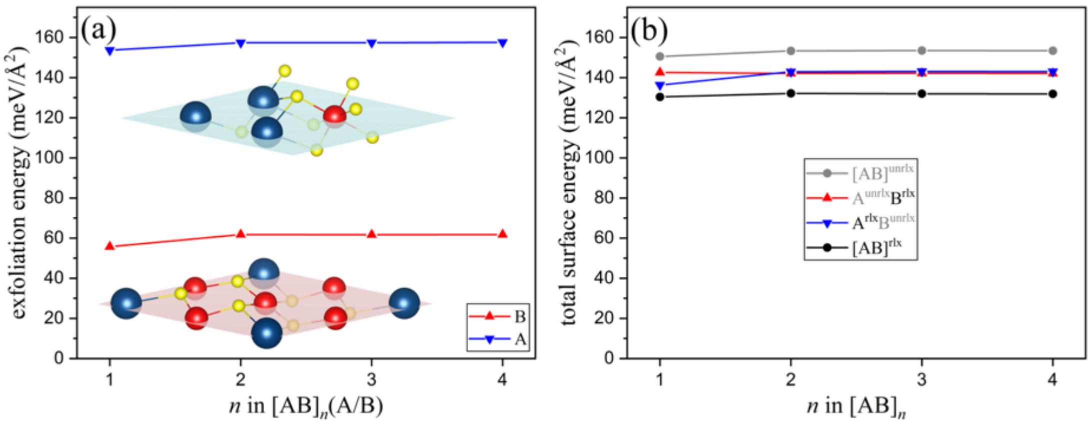

Supplementary Figure 4. a Exfoliation energy of A (Pr-rich) and B (Ir-rich) layers for peeling a single layer from the top of an $[AB]_n$ slab. **b** Total surface energy for $[AB]_n$ slab when the slab is unrelaxed (gray), only the B layer is relaxed (red), only the A layer is relaxed (blue), and the full slab is relaxed (black).

STM simulation of Pr- and Ir-rich surfaces

In order to simulate the constant-current STM images, we carried out an analysis of charge density isosurfaces for the A and B surface terminations. In each case, we optimized the slab at the PBE level with the settings detailed above. Using the charge density and wave functions from this run, a secondary calculation was performed to obtain the partial charge density for the states within ± 1 eV from the Fermi energy. Based on this data, we mapped the isosurfaces corresponding to various values of the charge density and obtained simulated STM images displayed in Supplementary Fig. 5.
An important observable in experimental STM images is the "step size" between surface regions defined by different terminations, such as the type-A and type-B layers. Corresponding values can be computed from the DFT charge density profiles and used to differentiate surface types observed in the experiment. Our DFT results in Supplementary Fig. 6 and Supplementary Table 1 show consistent values of the calculated height steps for a wide range of currents in constant-current STM imaging: 3.44 Å and 2.65 Å

for going from layer A to B and from layer B to A, respectively. Taking into account the grid size used to output the charge density in VASP and the variations in the metal atom positions within layers, we estimated the numerical error in the step height evaluations to be 0.12 Å. As discussed in the main text, this information turned out to be in excellent agreement with the experimental measurements and allowed us to identify conclusively plateaus with Pr- and Ir-rich terminations.

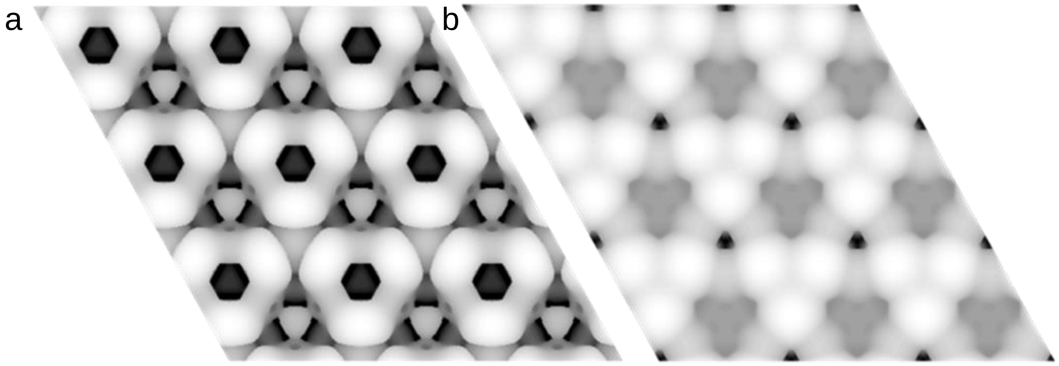

Supplementary Figure 5. Simulated constant-current STM images for the **a** Pr-rich and **b** Ir-rich surfaces. The images demonstrate the relative depth of the 0.029 e Å^{-3} charge density iso-surface. Darker color indicates a deeper positioning of the charge density iso-surface.

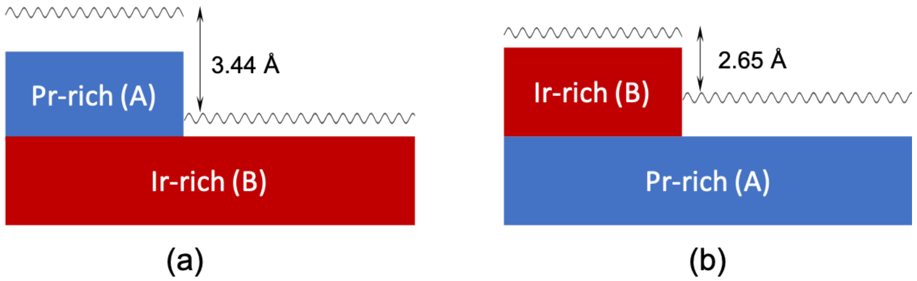

Supplementary Figure 6. Schematic of the average step size for a given value of the charge density going from **a** layer A to B; and **b** layer B to A. The values were obtained from the analysis of the charge density profile as detailed in Table 1.

charge density (e Å^{-3})	structures and step heights (Å)	
	A to B	B to A
0.364	3.44	2.65
0.675	3.44	2.65
0.965	3.45	2.64

Supplementary Table 1. Step size of the charge density going from layer A to B and vice versa. The values were tabulated for three different values of the charge density obtained in our DFT calculations.

Supplementary Note 3

We examine here whether the phase separation of the electronic states is related to the different surface terminations of the sample. To do this, we examine (in Supplementary Fig. 7a) a topography which exhibits sub-unit cell islands. These islands are a different termination from the rest of the topograph, meaning that we have both Pr-rich and Ir-rich surfaces showing here. In Supplementary Fig. 7c we show the Fourier transform of the topograph in (a), revealing the six Bragg peaks which correspond to the atomic Kagome structure in the sample. Supplementary Fig. 7d shows a linecut across the line shown in (a), revealing the island heights are less than a unit cell, and correspond to the Ir-rich surface termination as compared to our DFT calculations. To examine the possible relation of this to the phase separation, we also take a conductance map (Supplementary Fig. 7b) on the same area as the topograph in (a) at -14 meV. This map shows the electronic inhomogeneity and phase separation which we have been examining. Supplementary Fig. 7e shows the cross correlation between the topograph in (a) and the conductance maps at energies between -100 meV and +100 meV. The maximum value of correlation is 0.08, which means that at all the energies the correlation is negligible and there is no relation between the different surface terminations and the phase separation. Thus, the phase separation doesn't originate from a tunneling sensitivity to different surface terminations.

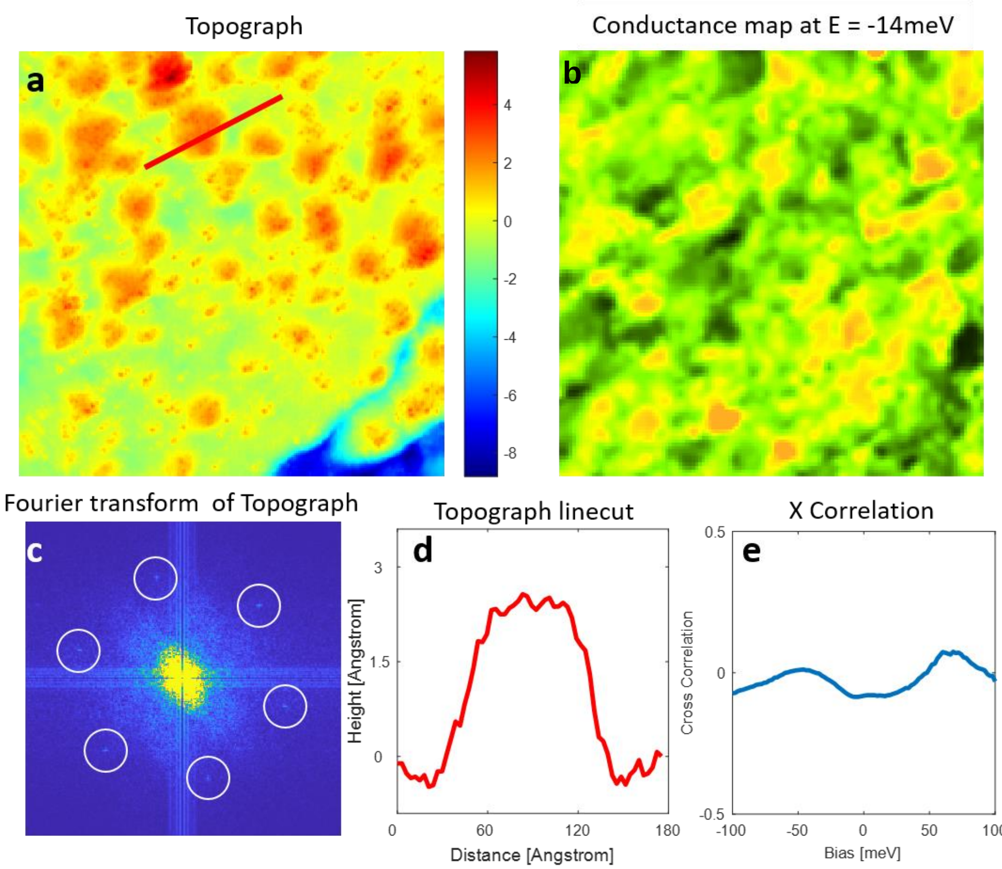

Supplementary Figure 7. a Topograph of a 65 nm area taken at 33 K. We see here two different surfaces. **b** Conductance map taken on the same area as the topograph in (a) and at an energy of -14 meV (resonance energy), which shows the electronic inhomogeneity and phase separation. **c** The Fourier transform of the topograph shows the Bragg peaks corresponding to the Kagome atomic structure. **d** A linecut of the red line in (a) showing that the surface has sub-unit cell islands, indicating the presence of both Pr-rich and Ir-rich surfaces. **e** The cross correlation of the topograph in (a) and the conductance maps at energies from -100 meV to 100 meV, revealing no correlation between them for all energies.

Supplementary Note 4

One base approximation of STM is that the conductance is roughly uniform over the measuring surface; this ensures that the conductance is proportional to the LDOS. This is enforced by maintaining the measured current above or below the Fermi energy at a constant value. In the case of $Pr_2Ir_2O_7$, however, this approximation breaks down, leading to an arbitrary scaling of spectra from one point to another on the surface. This renders the results of unsupervised machine learning unintelligible with unphysical bias.

The setpoint effect results in arbitrary modulations of the data samples

$$x_n(r) = \lambda(r) {x_n}^{physical}(r)$$

where the x_n are D-dimensional data vectors and $\lambda(r)$ accounts for an unknown setpoint scaling. To remove the unphysical modulation caused by the setpoint effect, each spectra was re-scaled to have unit norm in data-space upon interaction with the K-means algorithm. This is a bit different from the typical data science pre-process such as standardization, in which each of the D elements per sample has zero mean and unit standard deviation. We make this choice of normalization to remove the overall modulation due to the form of the setpoint effect. Following this clustering, all spectra are mapped back to their unnormalized form.

This input data varies from being on a 64 by 64 grid to a 256 by 256 grid, with spectra sampled at either 101 or 11 energies, respectively.

All K-means calculations were implemented via SciKit-Learn's software package[26], written in Python 3.7, and coded in the Jupyter environment[27]. We now give a brief review of the K-means framework.

The K-means algorithm sorts *N* D-dimensional samples, x_{nd}, into *K* clusters by separation distance in data-space. The distortion measure

$$J = \sum_n \sum_k r_{nk} \|x_n - \mu_k\|^2$$

gives the degree to which the clusters represent the original data. While in this paper we used D-dimensional Euclidean distance for our kernel, other options are certainly worth exploring in the future. The r_{nk} are binary indicator variables which assign the nth sample to the kth cluster: $r_{nk} = 1$ if $k = argmin_j$ $||x_n - \mu_j||^2$ and $k = 0$ otherwise; the μ_k are the cluster centers (means). We would like to determine the r_{nk}^0 and μ_k^0 which minimize the distortion measure[28].

This can be done iteratively, first by picking some initial values of the μ_k, often randomly assigned. Each iteration that follows has two phases: first assign each sample to the cluster with the most proximal mean, then recalculate the mean of each cluster. That is, we minimize J with respect to the r_{nk} with the μ_k fixed, then minimize J with respect to μ_k with the r_{nk} fixed. Iteration is continued until convergence is obtained and there is no change in cluster assignment; convergence is guaranteed, though convergence to a global minimum is not[28].

Phase 1: $r_{nk} = \begin{cases} 1 \; if \; k = argmin_j \|x_n - \mu_k\|^2 \\ 0 \; otherwise \end{cases}$; with μ_k fixed.

Phase 2: $\mu_k = \frac{\sum_n r_{nk} x_n}{\sum_n r_{nk}}$; with r_{nk} fixed.

We derive the equation used for Phase 2 from: $\frac{dJ}{d\mu_k} = 2\sum_n r_{nk}(x_n - \mu_k) = 0$.[28]

The output of the K-means algorithm is a pre-determined number of clusters into which all the inputted spectra are sorted. We visualize these clusters in a cluster map, which shows the real-space distributions of each cluster. We also examine the means of each cluster.

The input to the K-means algorithm was purely spectra, with no spatial information. However, the output of the algorithm shows a strong tendency of the spectral clusters to define spatial clusters, which we explore with the pair connectivity function.

Supplementary Note 5

We compare the results of the K-means calculations, which result in a cluster map (Supplementary Fig. 8a), with the corresponding conductance map from which it was formed (Supplementary Fig. 8b). We see a strong correlation for energies near the resonance (the -20 meV energy is shown in Supplementary Fig. 8b). We observe this correlation both visually, as many of the clusters in each map clearly match up, as well as by calculating the correlation between the two maps. We plot this correlation as a function of conductance map energy (inset of Supplementary Fig. 8b).

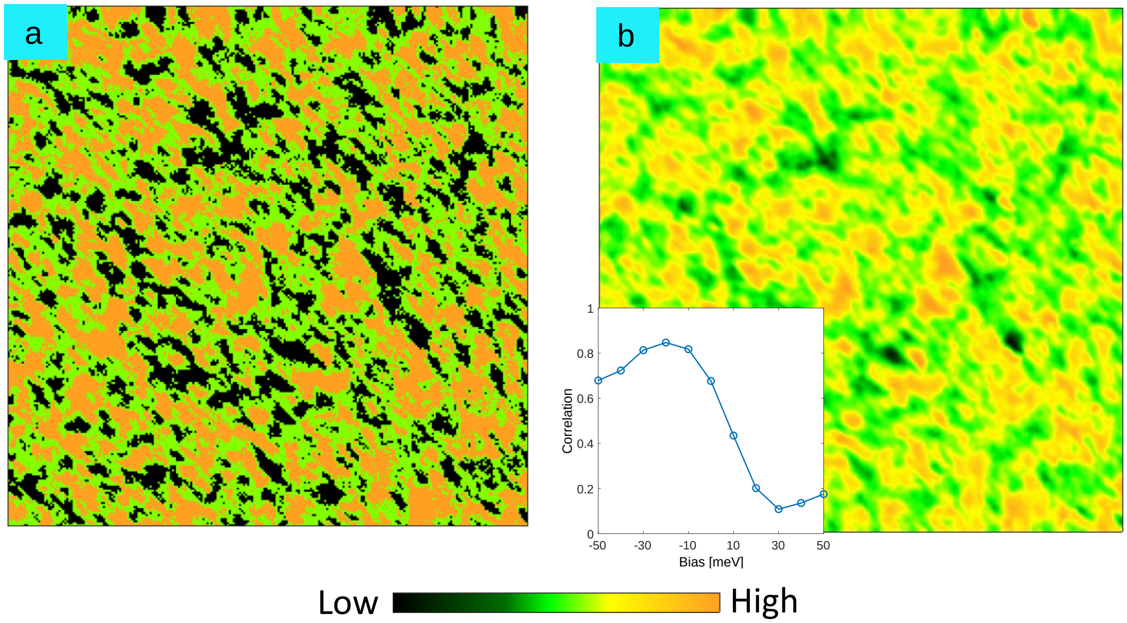

Supplementary Figure 8. a A 3-cluster K-means map. **b** A 256-pixel conductance map on which the K-means algorithm in (a) was run. This map shows the -20 meV energy. Even by eye there is clearly a strong correlation between the conductance map and its corresponding K-means cluster map. The correlation is calculated to be 85%.

We see that there is a strong correlation at biases below the Fermi energy, with the strongest correlations being around -10 to -30 meV. Thus, the K-means cluster maps are analogous to the STM conductance maps at energies around -20meV. The fact that the correlation drops for energies away from -20 meV is partially related to the STM set-point effect.

Supplementary Note 6

We show in Supplementary Fig. 9 a set of conductance maps at energies from -50 meV to +50 meV, at 33 K corresponding to the same field of view as Figure 1c and Figure 4a in the main manuscript. Underneath these conductance maps we show the Fourier transform of each map. The Fourier transforms all shows the six Bragg peaks which we expect from a Kagome lattice. Based on the six-fold symmetry we have taken the Fourier transforms and symmetrized them in order to enhance the images; the symmetrized Fourier transforms are shown here. We can clearly see the six Bragg peaks, and a central peak at q = 0 resulting from electronic inhomogeneity. We do not see any QPI signal with an energy dependence or any dispersion of a signal.

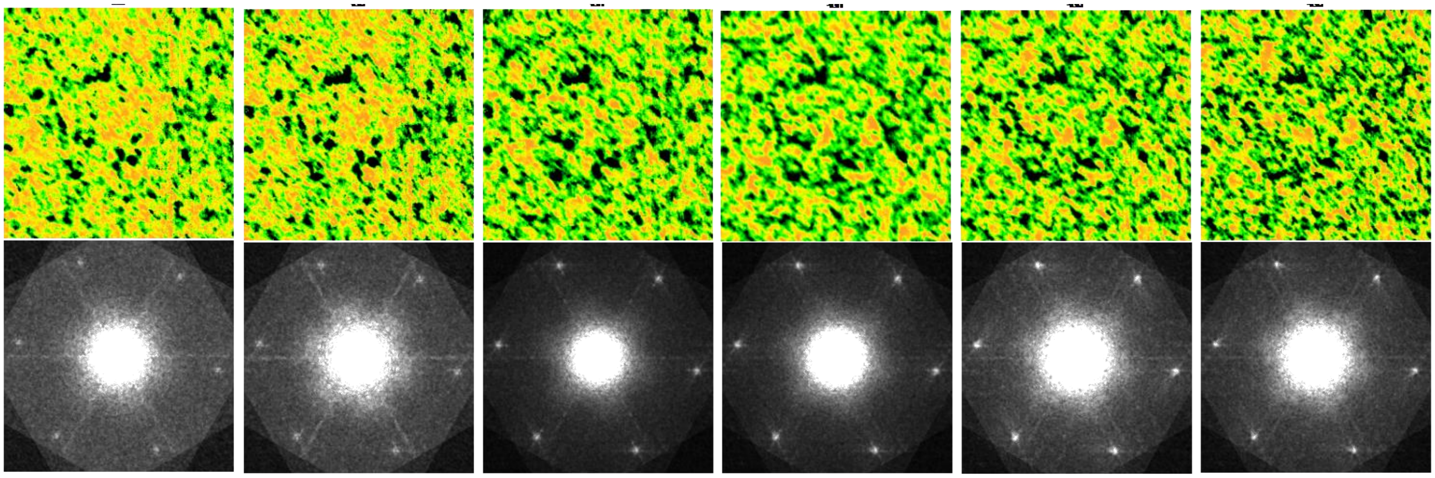

Supplementary Figure 9. Energy dependent conductance maps and their corresponding Fourier transforms measured at 33 K. Energies from left to right: 50 meV, 30 meV, 10 meV, -10 meV, -30 meV, -50 meV. The Fourier transforms are symmetrized.

Supplementary Note 7

To fit the resonance in the spectra, we use the Fano lineshape equation, which models the Kondo resonance. The Fano equation is given as

$$\frac{dI}{dV} \sim A \frac{(\frac{V-E}{\Gamma}+q)^2}{1+(\frac{V-E}{\Gamma})^2} + C + d * V$$

where E is the energy of the resonance, Γ is the width of the resonance (half width at half max), A is related to the amplitude of the resonance, C + d*V provides a linear background, V is the applied bias, and q is the asymmetry parameter of the Fano equation.

We start off by examining the average spectra of each of the three clusters. We know that the spectra which exhibit the resonance that we want to examine come from one of the three clusters shown in the K-means cluster maps. We then binarize the cluster maps so that the cluster which exhibits the resonance is separated from the two clusters which don't have this resonance (Supplementary Fig. 10).

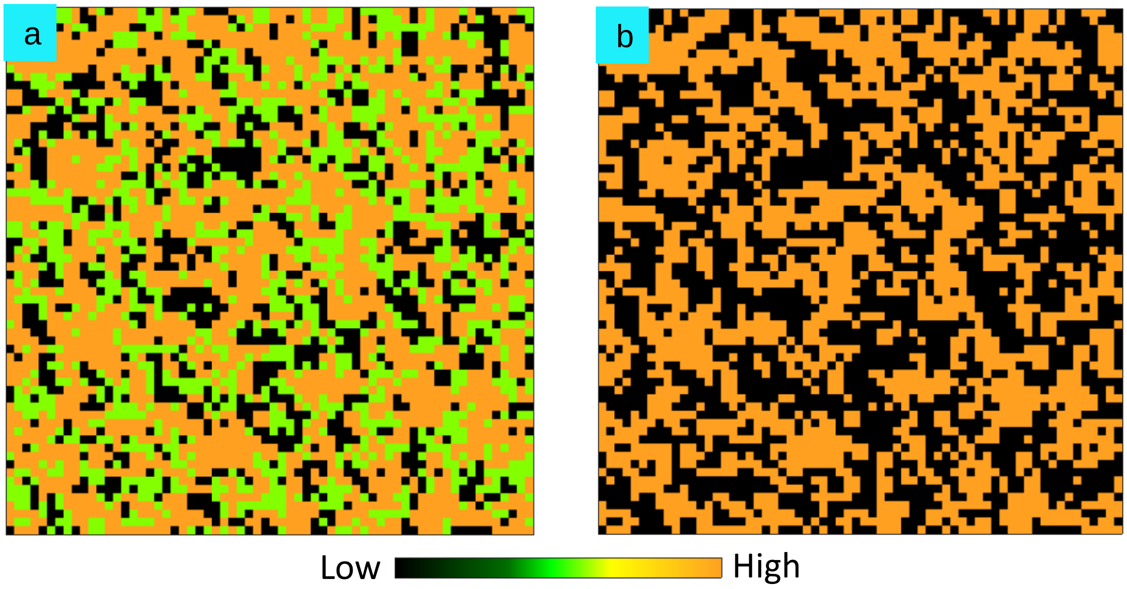

Supplementary Figure 10. a A 3-cluster K-means map. The orange corresponds to the areas with the Kondo resonance which we study. **b** The K-means map in (a) binarized; the orange is the resonance areas, and the black is non-resonance areas.

We then fit spectra from the cluster showing the resonance; we do this because the Fano lineshape model is based on the existence of a resonance. Trying to fit the model to a spectrum which doesn't have this resonance is a meaningless exercise.

When we examine some spectra from this resonance cluster, we see that the magnitude of the resonance continuously weakens and often disappears near the boundaries of the cluster (Supplementary Fig. 11). This occurs as a result of the continuous phase transition, which happens when going from one domain to the other.

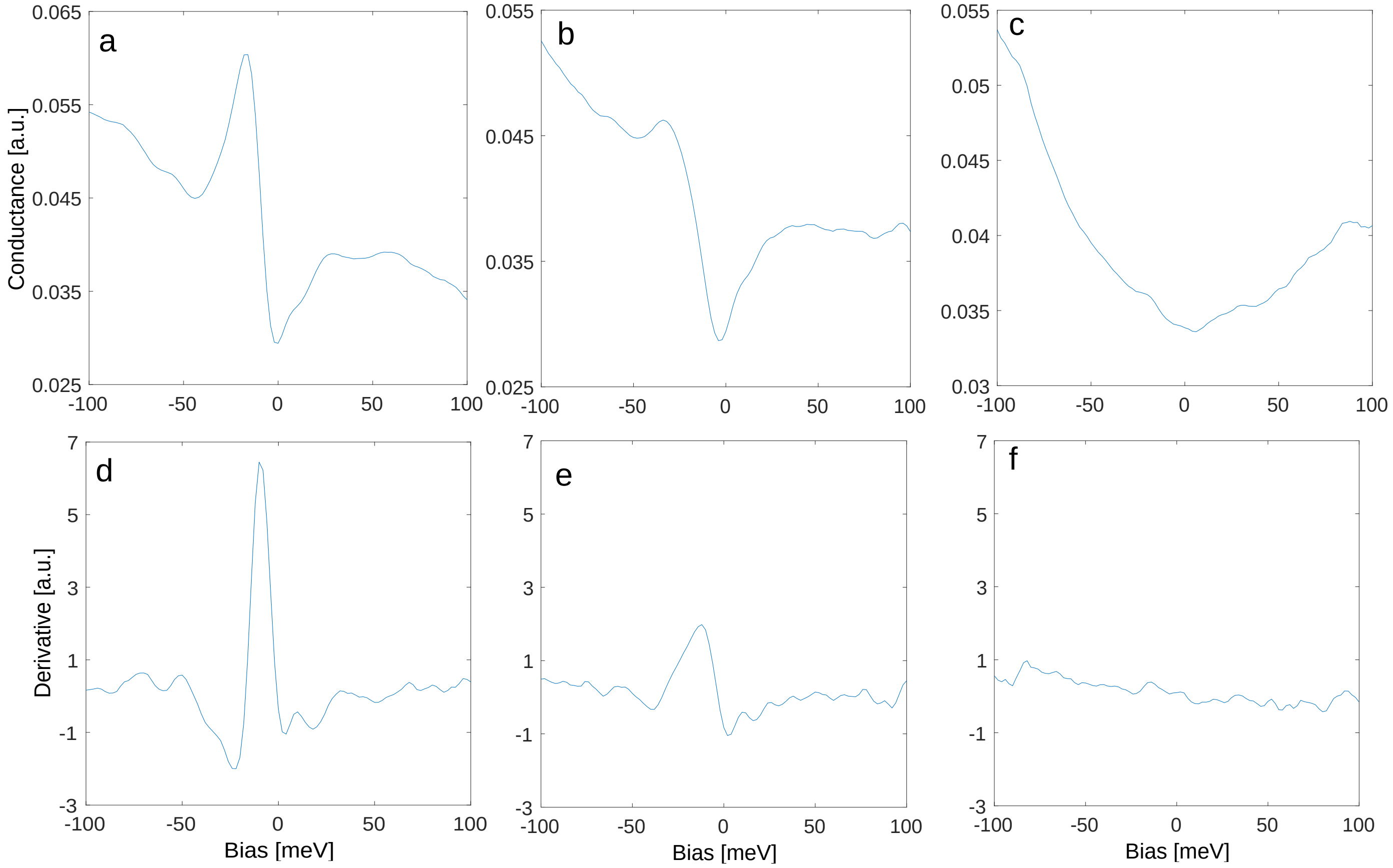

Supplementary Figure 11. a-c Three spectra taken from the cluster whose average shows the Kondo resonance. Clearly **a** the first spectrum strongly shows the resonance; **b** the second, taken from a domain edge, shows the resonance quite weakly, while **c** the third does not show the resonance. **d-f** Derivative of the spectra in (a)-(c), respectively. Clearly, the derivatives of the spectrum which show the resonance are quite different than the derivative of the spectra without.

In order to further remove spectra which do not have the resonance, we look at the first derivative of the spectra around the region where the resonance is seen; the resonance occurs around -10 meV, so we examine a range of roughly -40 meV to 10 meV. When the resonance is present, the maximum value of the derivative is much larger than the average value of the derivative (Supplementary Fig. 11). Using this fact, we remove from our fitting any spectra where the maximum is not significantly (usually 5 to 10 times as much) larger than the average. Supplementary Fig. 12 shows the results of this filtering: we see the map from Supplementary Fig. 10b, and now the orange cluster is a more limited set of resonance spectra. This is the set to which we fit the resonance model. Additionally, many of the spectra which we remove this way, marked in green, are on the edges of domains, which indicates that the resonance is likely weaker on domain edges than at the center of the Kondo resonance domains.

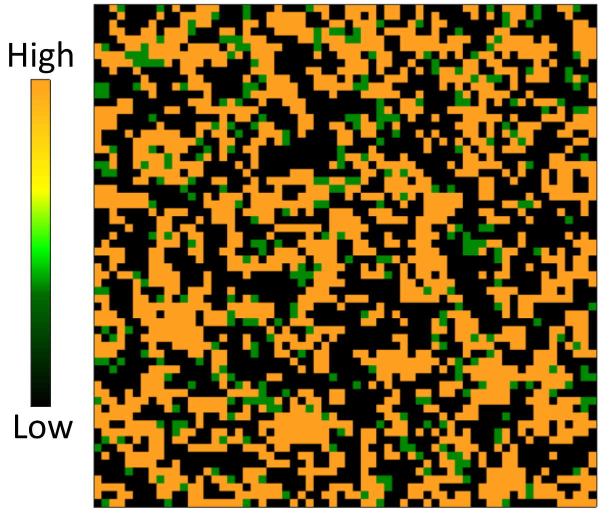

Supplementary Figure 12. The map from Supplementary Fig. 10b, where the orange corresponds to the resonance areas and the black corresponds to the non-resonance areas. Now, the resonance clusters are filtered to remove as many non-resonance spectra as possible; these newly removed spectra are shown in green. Most of these are on domain edges. The remaining orange spectra are the ones we fit to the Fano lineshape.

We now fit our filtered data using the Fano lineshape between -40 meV to 10 meV to accurately capture the resonance peak. Each spectrum is fit to the Fano lineshape with a simple linear background. Supplementary Fig. 13 shows a representative spectrum along with its fit in the -40 meV to 10 meV range. From each fit we extract histograms of the various parameters in the Fano equation.

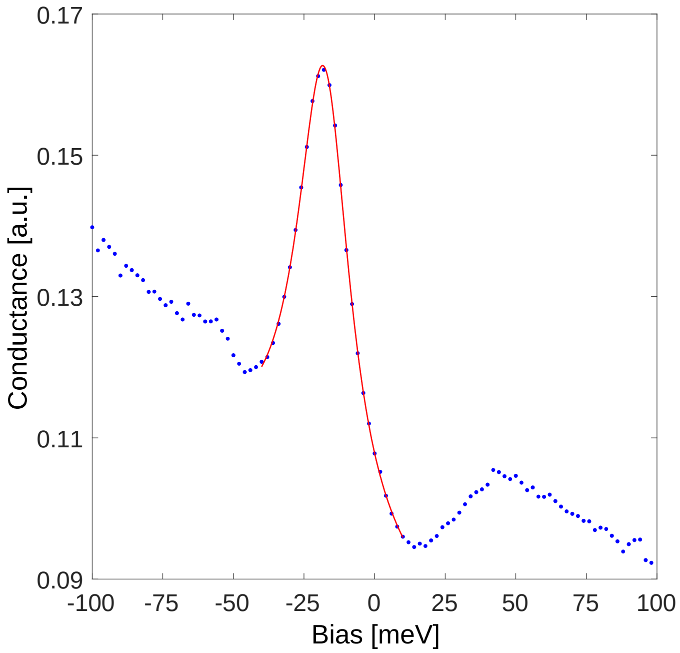

Supplementary Figure 13. A spectrum (blue markers) showing the Kondo resonance along with the fit (red line) of the resonance to the Fano lineshape.

We perform this analysis for K-means cluster maps formed from data at both 10 K and 33 K. We represent each of the parameters of interest, Γ, E, and q, in the form of a histogram; we additionally calculate the amplitude, given as $A(1 + q^2)$. Supplementary Fig. 14 shows the histograms of these four quantities for both 10 K and 33 K. For these eight sets of data we calculate both the average value and the standard deviation. This standard deviation is calculated for each based off of the Gaussian which each histogram shows.

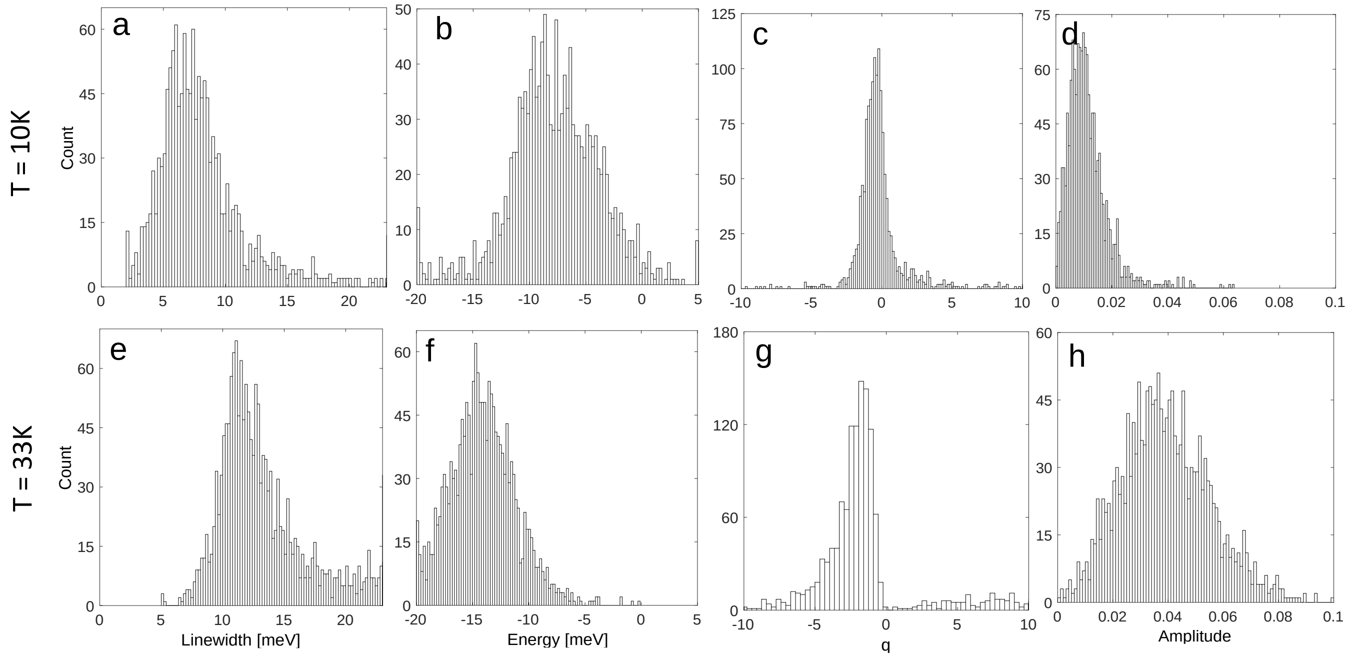

Supplementary Figure 14. a-d 10 K data. **a** shows a histogram of the linewidth with an average of 7.4 ± 2.2 meV. This results in a Kondo temperature of 56 K. **b** shows a histogram of the energy with an average of -7.6 ± 3.2 meV. **c** shows a histogram of the asymmetry parameter q with an average of -0.4 ± 0.9. **d** shows a histogram of the amplitude with an average of 0.010 ± 0.006. **e-h** 33 K data. **e** shows a histogram of the linewidth with an average of 11.8 ± 1.9 meV. This results in a Kondo temperature of 64 K. **f** shows a histogram of the energy with an average of -13.9 ± 2.3 meV. **g** shows a histogram of the asymmetry parameter q with an average of -2.2 ±1.0. **h** shows a histogram of the amplitude with an average of 0.04 ± 0.01. All errors are calculated as the standard deviation of the Gaussian shown.

Using the average value of the linewidth of this Kondo resonance, and a relationship between the linewidth and the Kondo temperature,

$$\Gamma = \sqrt{(\pi k_B T)^2 + 2(k_B T_K)^2}$$

we calculate the Kondo temperature. For the 10 K dataset we get a Kondo temperature of 56 $\pm$ 20 K; for the 33 K dataset we get a Kondo temperature of 64 $\pm$ 20 K. Since we form our range of Kondo temperatures from the standard deviation of the linewidths, the T_K and the error here correspond to the range of Kondo temperatures through the entire Gaussian distribution.

Additionally, we attempt to perform this fitting analysis for K-means cluster maps from 45 K data. As expected from the average spectra resulting from the 45 K cluster maps, the spectra at this temperature exhibit a very weak resonance. Furthermore, since the background is unknown, it makes the fitting less reliable.

Supplementary Note 8

Here we compare the theoretical calculation of the DOS for a quadratic band touching to the experimental data obtained. Supplementary Fig. 15a shows the calculation of the DOS based on having two quadratic bands touching at the Fermi energy. The quadratic bands both above and below the Fermi energy are taken as identical. The units of the DOS are arbitrary. The DOS shows a partial gap structure with a square root energy dependence, which becomes thermally broadened at higher temperatures. In Supplementary Fig. 15b we have a similar picture, however we now have one adjustable parameter which allows modification of the DOS magnitude of one band. Supplementary Fig. 15c, we show the experimentally obtained data from the dark green K-means clusters, at both 10 K and 33 K. The figure shows the spatial average of all dark green spectra in the dataset. These show a similar DOS profile to the theoretical prediction. The minima of the spectra are within ± 2 meV of the Fermi energy, indicating the charge neutrality and therefore the stoichiometry of the samples.

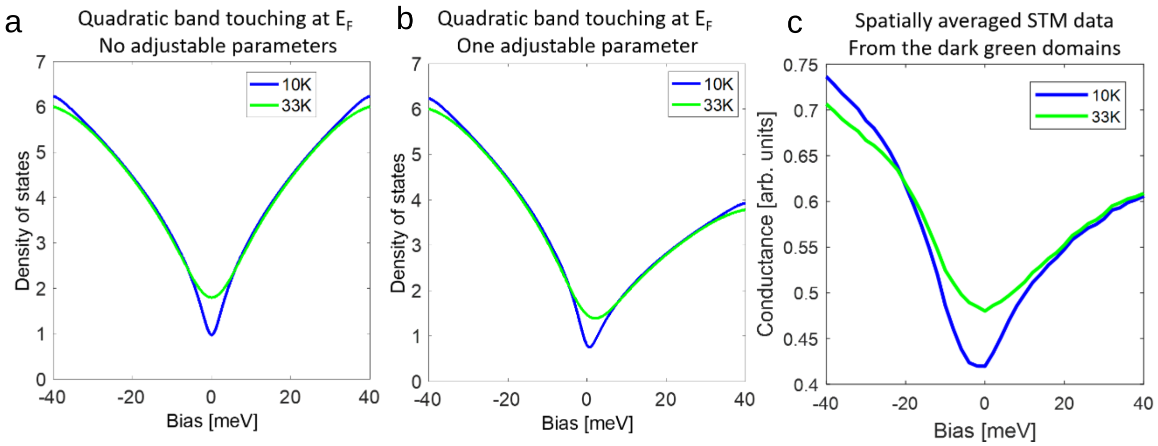

Supplementary Figure 15. Density of states from a quadratic bands touching at E_F with **a** no adjustable parameters, and **b** with one adjustable parameter. **c** Spatially averaged STM data, from the dark green domains. Data are shown for both 10 K and 33 K. The vertical axes have arbitrary units.

Supplementary Note 9

We now examine histograms of conductance maps for data at both 10 K and 33 K, specifically the conductance maps at the energy where we observe the resonance peak, which happens around -10 to -20 meV. In Supplementary Fig. 16a we show a histogram of all conductance values from a conductance map taken at 10 K and at an energy of -10 meV. Similarly, in Supplementary Fig. 16c we show a histogram of all conductance values from a map taken at 33 K and also at an energy of -10 meV.

For each temperature, we start with the 2-cluster K-means map (binarized map). Using the two clusters from K-means we split the conductance map into the same two clusters. We then take each of the two clusters individually and create a histogram from the conductance values which form each cluster. Plotted in Supplementary Fig. 16b and 16d we show the overall histogram (as seen in Supplementary Fig. 16a and 16c) along with the two clusters' individual histograms.

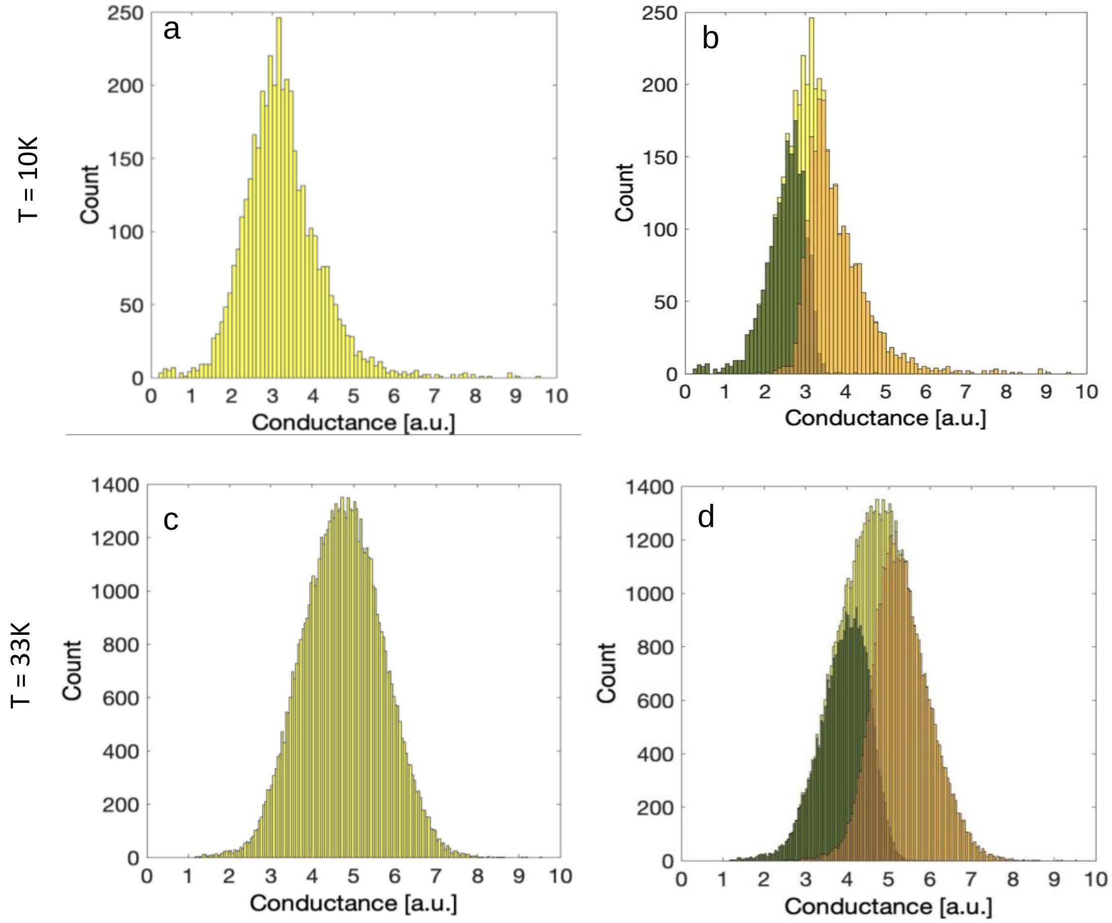

Supplementary Figure 16. a,c show the histograms of the entire set of conductance values taken from the conductance map at the resonance energy. **a** is at 10 K and is taken from a -10 meV conductance map and **c** is at 33 K and is also taken from a -10 meV conductance map. **b,d** show the histograms in (a),(c), respectively, with the histograms of the two clusters overlaid.

The histograms in Supplementary Fig. 16 a,c allow us to look at the density of states (the value of the conductance) near the resonance, which is how we define our clusters. We see in the histograms the formation of a single Gaussian, rather than a binomial distribution, which indicates we have a continuous transition from the areas of low conductance to areas of high conductance.

Additionally, when we plot the density of states of the two clusters individually, we see that rather than being binomial there is a large overlap between the two, which supports the conclusion that one cluster transitions continuously into the other.

Supplementary Note 10

When analyzing the geometric clusters, one of the important factors to consider is the accuracy of those clusters. The resolution of the conductance maps taken has a major effect on how accurately we see these clusters. With too low of a resolution, smaller clusters are not seen, and this greatly affects the analysis. Seen in Supplementary Fig. 17a,b are two conductance maps, both taken on the same area of 65 nm. The first map is taken with a 64x64 pixel resolution, while the second is taken with 256x256 pixel resolution.

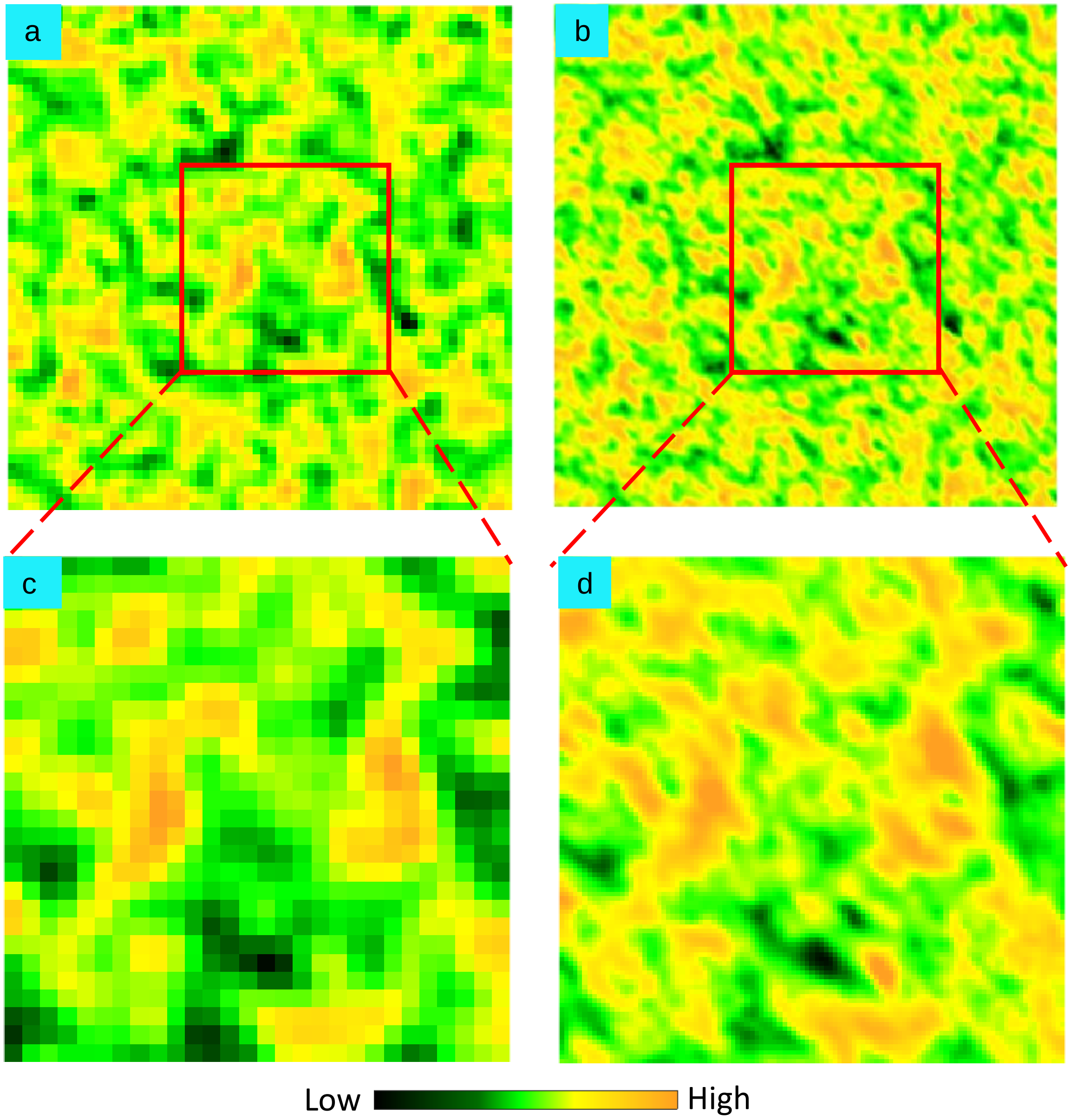

Supplementary Figure 17. a A conductance map taken with 64x64 pixel resolution. **b** A higher resolution 256x256 pixel conductance map taken on the same area as the map in (a). **c** Zoomed-in image of the red box in (a). **d** Zoomed-in image of the red box in (b). Much more detail is clearly evident.

Looking at the map overall, many of the main clusters are seen in both maps. However, the details within these larger clusters are quite different between maps. This is because one pixel of the lower resolution map is equivalent to 16 pixels in the higher resolution map, thus allowing us to see much more detail. Supplementary Fig. 17c,d shows zoomed-in images from the red boxes in Supplementary Fig. 17a,b, allowing us to clearly see the difference in detail between the two resolutions.

The areas and boundaries of each cluster are much more detailed, clusters which appeared to be connected can be seen to actually be separate, and smaller clusters that can't be visualized in the lower resolution map can be seen in the higher resolution map. This improved resolution of clusters and their boundaries is essential for any proper geometric cluster analysis.

Supplementary Note 11

As discussed in the main paper, we use a K-means cluster map for the analysis of the domains. In Supplementary Fig. 18 and 19 we first compare the conductance map and K-means cluster map of the same data set. Supplementary Fig. 18a and 19a show the conductance map and K-means maps, respectively, of the same dataset. Supplementary Fig. 18b shows the spectra taken from high and low areas of conductance from the conductance map in Supplementary Fig. 18a; Supplementary Fig. 19b shows the average spectra from each of the two clusters shown in the K-means map in Supplementary Fig. 19a.

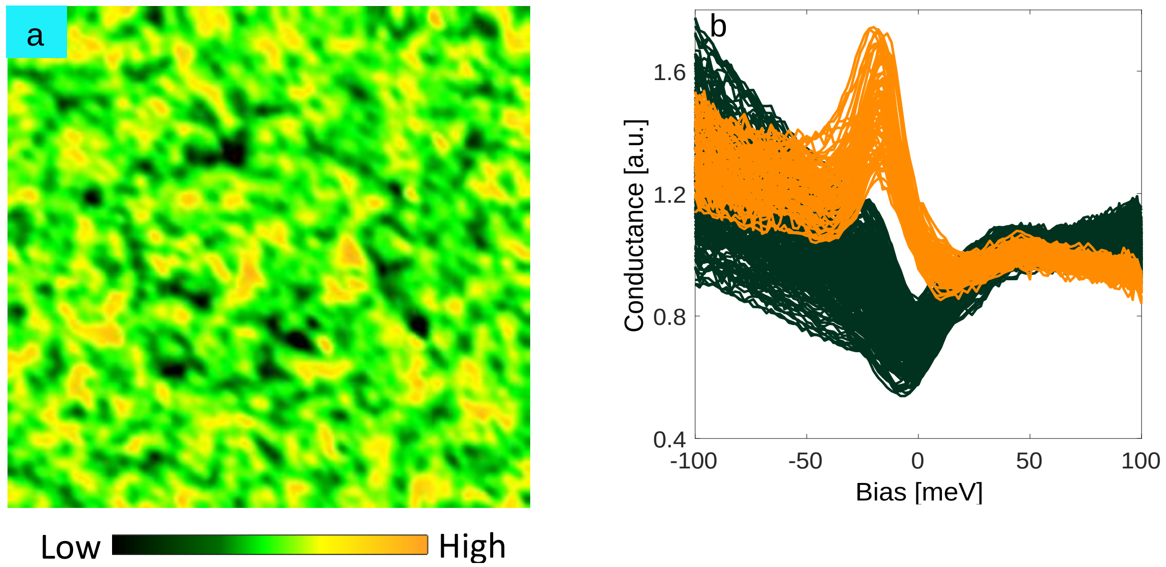

Supplementary Figure 18. A Conductance map taken with high resolution. **b** Spectra from areas of high (orange) and low (dark green) conductance.

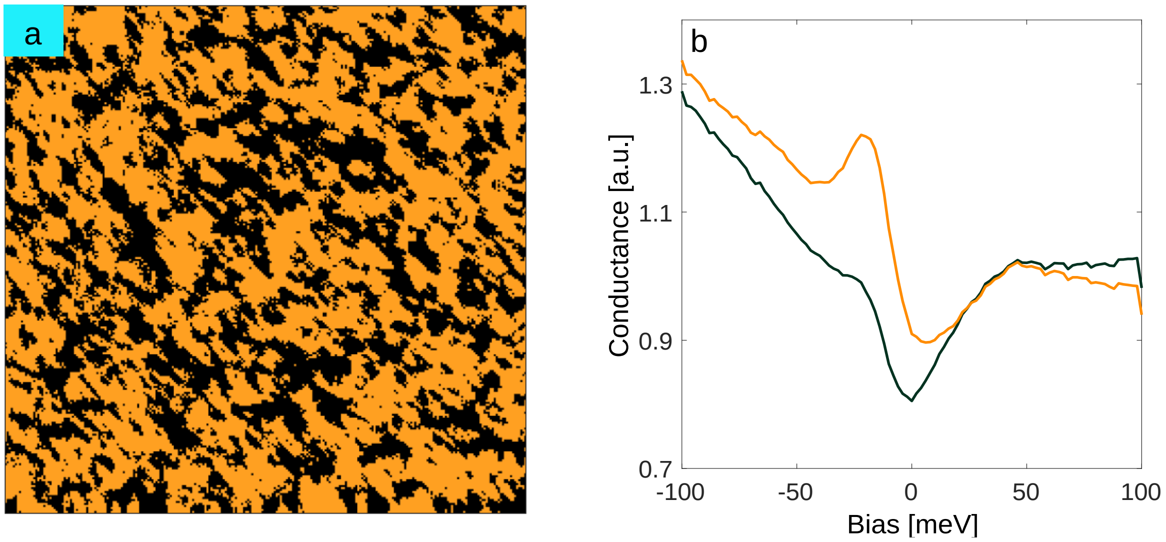

Supplementary Figure 19. a K-means cluster map from the dataset in Supplementary Fig. 18a. **b** Average spectra from the two clusters shown in (a). The orange spectrum correspond to the orange clusters and the black spectrum to the black clusters.

The significant conclusion from this comparison is that the K-means map separates the spectra into two clusters corresponding to high and low conductance areas in the conductance map, and thus are used to form the domains which we investigate. Using K-means rather than the conductance map for determining domains has the additional benefit of clearly defining the boundaries of each cluster; unlike in the conductance map, where there is no clear way to determine the boundary of each cluster.

For the following analysis, we use two methods of limiting the clusters in the K-means map. The first way is to remove clusters of overly small and overly large area; this is shown in Supplementary Fig. 20b. The second method is to additionally remove clusters which cross the boundary of the image; this is shown in Supplementary Fig. 20c. There are two reasons why we want to limit the cluster areas. First, small clusters less than 5 pixels are just as likely to be random errors as they are to be a real cluster, so we eliminate them. Secondly, clusters with areas larger than 2000 pixels create much more variability and error in the fits, and often appear to be many clusters that are connected by just a single pixel, so we eliminate these as well.

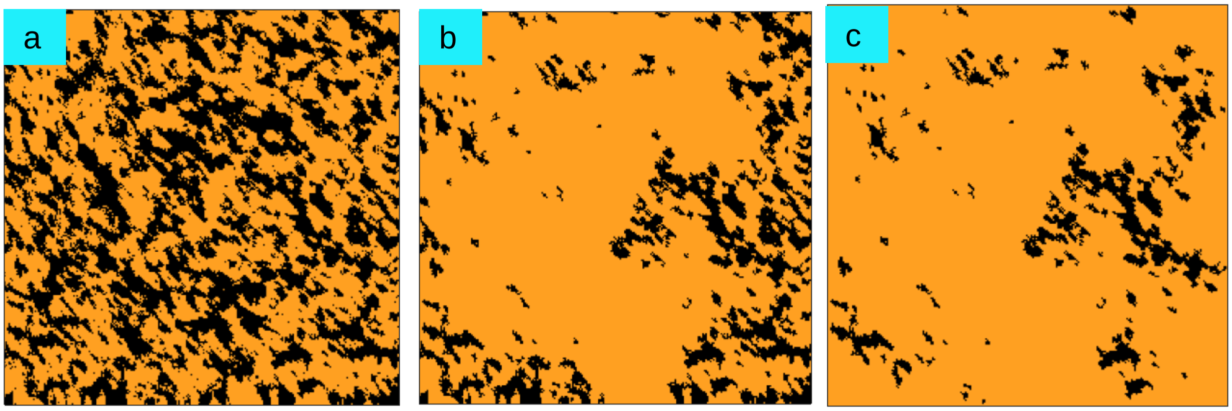

Supplementary Figure 20. a K-means cluster map. **b** Map after having removed clusters with areas smaller than 5 pixels and larger than 2000 pixels. **c** Map after having removed both small and large clusters and additionally removing clusters crossing the boundary of the image.

While analyzing these domains we find that there is little difference between using the data in Supplementary Fig. 20b and Supplementary Fig. 20c. Thus we decide to limit our data by removing small and large clusters but leaving the boundary-crossing clusters intact.

The way that we implement this data limitation varies depending on the analysis method. For both the box-counting method and for analyzing the cluster area distribution, the input to our algorithms is the dataset where we have already removed the small and large area clusters (seen in Supplementary Fig. 20b). For the geometric domain analysis relating area, perimeter, and radius of gyration, we provide the full data (Supplementary Fig. 20a) and we then exclude the points from our fit which correspond to the large and small pixel areas in the dataset (excluded points shown by filled in circles in main paper figure).

The box-counting method is known to theoretically measure the dimension of fractal patterns. We use this idea to measure the fractal dimension of the clusters. We divide the 256-pixel map into a mesh of boxes. We vary this box size from 1 pixel up to 12 pixels, and for each different box size we count the number of boxes N that include part of a cluster. We then fit this data to the scale-invariant power law $N \sim (\frac{1}{boxsize})^D$, using the least square method, to find the fractal dimension D = 1.49 ± 0.02.

Additionally, we analyze the fractal properties of these clusters using geometric domain methods. We apply an image region analysis software which looks at all the clusters and tells us both the area and perimeter of each. From these measurements we also calculate the radius of gyration, $R_g = \sqrt{\langle (r - \langle r \rangle)^2 \rangle}$, where r is summed over all the points in each individual cluster, for each cluster in the map.

A scale-invariant property similar to that of the box-counting method appears in the cluster area distribution: the cluster area A and the number of clusters N(A) follow the power law $N(A) \sim A^{\tau}$. We take all the areas of the clusters, logarithmically bin them, count and normalize the numbers of clusters with areas within the logarithmic bins, and fit this data to the power law; we then get a Fisher exponent of τ = 1.62 ± 0.16.

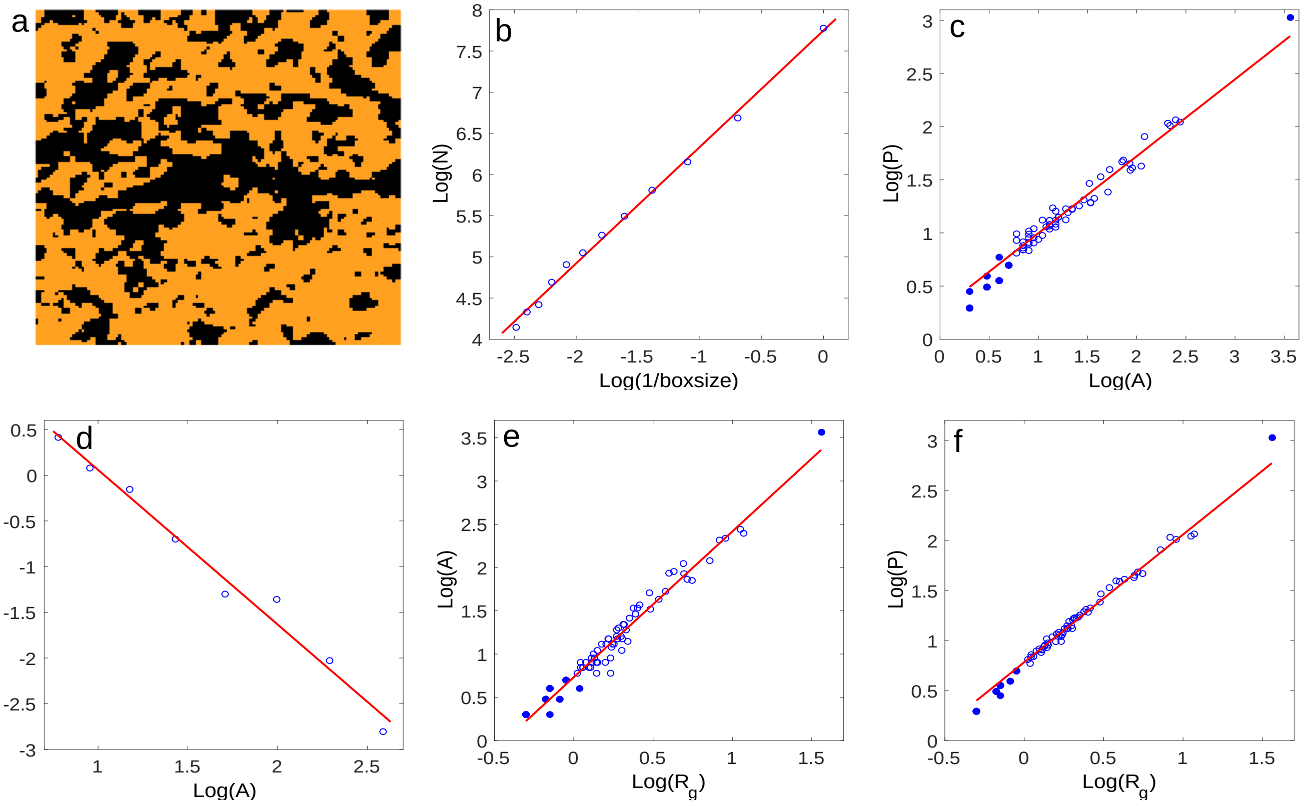

Supplementary Figure 21. a K-means cluster map (50 nm) forced using two clusters. The experimental data was acquired with a 64x64 pixel resolution and extrapolated into a 128x128pixels. The black clusters were used for the fractal analysis. The data is taken at 10 K. **b** The fractal dimension analysis using a box-counting method resulting in a fractal dimension D = 1.41 ± 0.06. The data points are fitted by the least square method to the power law $N = \left(\frac{1}{boxsize}\right)^D$ where N denotes the number of boxes of clusters. **c** Perimeter vs area of the clusters. Filled circles are excluded from the fit. Using $P = A^{D/2}$ gives a fractal dimension value of D = 1.45 ± 0.04; this agrees very well with the box-counting method. **d** Cluster area distribution with logarithmic binning, giving a Fisher exponent τ = 1.69 ± 0.23. **e** Utilizing the radius of gyration for each cluster, the equation $A = {R_g}^{d_v}$ gives a value of d_v = 1.69 ± 0.11. **f** Similarly using $P = {R_g}^{d_h}$ gives a value of d_h = 1.28 ± 0.04. These exponents are comparable to the those obtained in the main paper for the 33 K data.

Similar relationships exist relating the area and perimeter and relating the radius of gyration to the area and to the perimeter. Using $P = cA^{D/2}$ gives a fractal dimension value of D = 1.53 ± 0.03, using $A = {R_g}^{d_v}$ gives a value for the volume fractal dimension of d_v = 1.76 ± 0.06 and using $\mathrm{P} = {R_g}^{d_h}$ gives a value for the hull fractal dimension of d_h = 1.39 ± 0.03.

All of the errors that we give for the various critical exponents come from calculating the 95% confidence value for each of the fittings we perform.

We also analyze and obtain the similar parameters for a 10 K dataset (Supplementary Fig. 21). We can see that all of the exponents obtained here are quite similar to the values obtained from the previous dataset.

Supplementary Note 12

Without inputting any spatial information to the K-means calculation, the output spectral clusters manifest as spatially distinct domains. To understand the statistics of these spatial clusters, we use a tool of graph theory typical to the field of percolation called the pair connectivity function (PCF)[29–31], defined as $g_{conn}(r) = r^{-(d+2+\eta)}e^{-r/\xi}$. Using this we capture the spatial cluster size and near-transition power law behavior. Here d is the dimensionality, η is the connectivity, and ξ is the correlation length.

For surface measurements $d = 2$, so $g_{conn}(r) = r^{-\eta}e^{-r/\xi}$. To compute $g_{conn}(r)$ from our experimental data we turn to an equivalent quantity: the likelihood that two points are connected, given that they belong to the same spectral cluster, and that the points are a distance r apart. We know which points are in the same spectral clusters from the results of the k-means clustering. Once a sampling of $g_{conn}(r)$ has been constructed, the connectivity exponent and the correlation length are extracted by fitting this function to the extracted data.

To compute this likelihood of connection between two points, we sample the fraction

$$\frac{Number\ of\ connected\ pairs}{Number\ of\ pairs}$$

a thousand times at a given range. Repeating this for several values of r, we obtain the approximation

$$g_{conn}(r) = r^{-\eta}e^{-r/\xi} \approx \frac{Number\ of\ connected\ pairs\ sampled}{Number\ of\ pairs\ sampled}(r)$$

for each spectral cluster.

Supplementary Note 13

To understand the unique behaviour of the Kondo scale and its sensitivity to small perturbations of the chemical potential in the case of a quadratic band touching at the Fermi energy, we also consider, for comparison, a usual metallic system where the density of states is roughly a constant.

We model this case in terms of two degenerate conduction-electron bands, each having a semi-elliptic density of state $\rho(E) = \frac{2}{D^2\pi}\sqrt{D^2 - E^2}$, with μ not being too far away from $E = 0$.

Supplementary Fig. 22 plots the corresponding result, $T_K^0(\mu)/T_K^0(\mu_0 = -0.2D)$ as a function of μ. Compared with the quadratic band touching case, the Kondo scale is considerably less sensitive to the change in μ.

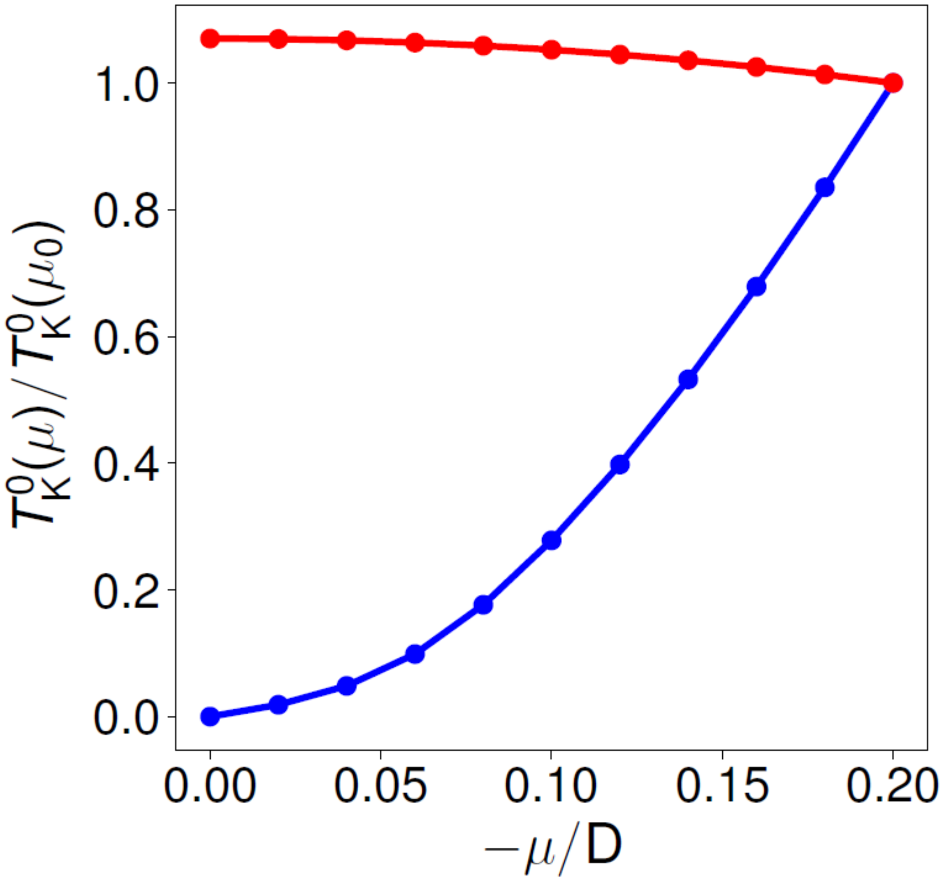

Supplementary Figure 22. Measure of the Kondo scale, $T_K^0(\mu)/T_K^0(\mu_0 = -0.2D)$, as a function of the chemical potential for a metallic band with roughly constant density of states (red data points) and for a quadratic band touching (blue data points).

Supplementary References

1. Yanagishima, D. & Maeno, Y. Metal-Nonmetal Changeover in Pyrochlore Iridates. *J. Phys. Soc. Japan* **70**, 2880–2883 (2001).
2. Machida, Y. *et al.* Crystalline electric field levels and magnetic properties of the metallic pyrochlore compound $Pr_2Ir_2O_7$. in *Journal of Physics and Chemistry of Solids* **66**, 1435–1437 (2005).
3. Nakatsuji, S. *et al.* Metallic Spin-Liquid Behavior of the Geometrically Frustrated Kondo Lattice $Pr_2Ir_2O_7$. *Phys. Rev. Lett.* **96**, 087204 (2006).
4. Machida, Y. *et al.* Unconventional Anomalous Hall Effect Enhanced by a Noncoplanar Spin Texture in the Frustrated Kondo Lattice $Pr_2Ir_2O_7$. *Phys. Rev. Lett.* **98**, 057203 (2007).
5. Matsuhira, K. *et al.* Metal-Insulator Transition in Pyrochlore Iridates $Ln_2Ir_2O_7$ (Ln = Nd, Sm, and Eu). *J. Phys. Soc. Japan* **76**, 043706 (2007).
6. MacLaughlin, D. E. *et al.* Muons and frustrated magnetism in $NiGa_2S_4$ and $Pr_2Ir_2O_7$. *J. Phys.: Conf. Ser.* **225**, 012031 (2010).
7. Matsuhira, K., Wakeshima, M., Hinatsu, Y. & Takagi, S. Metal-Insulator Transitions in Pyrochlore Oxides $Ln_2Ir_2O_7$. *J. Phys. Soc. Japan* **80**, 094701 (2011).
8. Ishii, F. *et al.* First-Principles Study on Cubic Pyrochlore Iridates $Y_2Ir_2O_7$ and $Pr_2Ir_2O_7$. *J. Phys. Soc. Japan* **84**, 073703 (2015).
9. Zhang, H., Haule, K. & Vanderbilt, D. Metal-Insulator Transition and Topological Properties of Pyrochlore Iridates. *Phys. Rev. Lett.* **118**, 026404 (2017).
10. Georges, A., Kotliar, G., Krauth, W. & Rozenberg, M. J. Dynamical mean-field theory of strongly correlated fermion systems and the limit of infinite dimensions. *Reviews of Modern Physics* **68**, 13–125 (1996).
11. Kotliar, G. *et al.* Electronic structure calculations with dynamical mean-field theory. *Rev. Mod. Phys.* **78**, 865–951 (2006).
12. Hohenberg, P. & Kohn, W. Inhomogeneous Electron Gas. *Phys. Rev.* **136**, B864–B871 (1964).
13. Kohn, W. & Sham, L. J. Self-Consistent Equations Including Exchange and Correlation Effects. *Phys. Rev.* **140**, A1133–A1138 (1965).
14. Perdew, J. P., Burke, K. & Ernzerhof, M. Generalized Gradient Approximation Made Simple. *Phys. Rev. Lett.* **77**, 3865–3868 (1996).
15. Liechtenstein, A. I., Anisimov, V. I. & Zaanen, J. Density-functional theory and strong interactions: Orbital ordering in Mott-Hubbard insulators. *Phys. Rev. B* **52**, R5467–R5470 (1995).
16. Dudarev, S. L., Botton, G. A., Savrasov, S. Y., Humphreys, C. J. & Sutton, A. P. Electron-energy-loss spectra and the structural stability of nickel oxide: An LSDA+U study. *Phys. Rev. B* **57**, 1505–1509 (1998).
17. Heyd, J., Scuseria, G. E. & Ernzerhof, M. Hybrid functionals based on a screened Coulomb potential. *J. Chem. Phys.* **118**, 8207–8215 (2003).
18. Langreth, D. C. & Mehl, M. J. Beyond the local-density approximation in calculations of ground-state electronic properties. *Phys. Rev. B* **28**, 1809–1834 (1983).
19. Blöchl, P. E. Projector augmented-wave method. *Phys. Rev. B* **50**, 17953–17979 (1994).
20. Kresse, G. & Hafner, J. *Ab initio* molecular dynamics for liquid metals. *Phys. Rev. B* **47**, 558–561 (1993).
21. Kresse, G. & Furthmüller, J. Efficient iterative schemes for *ab initio* total-energy calculations using a plane-wave basis set. *Phys. Rev. B* **54**, 11169–11186 (1996).
22. Monkhorst, H. J. & Pack, J. D. Special points for Brillouin-zone integrations. *Phys. Rev. B* **13**, 5188–5192 (1976).
23. Krukau, A. V., Vydrov, O. A., Izmaylov, A. F. & Scuseria, G. E. Influence of the exchange screening parameter on the performance of screened hybrid functionals. *J. Chem. Phys.* **125**, 224106 (2006).

24. Han, Y. *et al.* Surface energies, adhesion energies, and exfoliation energies relevant to copper-graphene and copper-graphite systems. *Surf. Sci.* **685**, 48–58 (2019).
25. Neugebauer, J. & Scheffler, M. Adsorbate-substrate and adsorbate-adsorbate interactions of Na and K adlayers on Al(111). *Phys. Rev. B* **46**, 16067–16080 (1992).
26. Pedregosa, F. *et al.* Scikit-learn: Machine Learning in Python. *J. Mach. Learn. Res.* **12**, 2825–2830 (2011).
27. Perez, F. & Granger, B. E. IPython: A System for Interactive Scientific Computing. *Comput. Sci. Eng.* **9**, 21–29 (2007).
28. Bishop, C. M. *Pattern Recognition and Machine Learning*. (Springer Science+Business Media LLC, New York, 2006).
29. Kadanoff, L. P. *Statistical Physics: Statics, Dynamics and Renormalization*. (World Scientific Publishing Company, Singapore, 2000).
30. Liu, S., Carlson, E. W. & Dahmen, K. A. Connecting Complex Electronic Pattern Formation to Critical Exponents. Preprint at https://arxiv.org/abs/1803.08485 (2018).
31. Li, J. *et al.* Scale-invariant magnetic textures in the strongly correlated oxide $NdNiO_3$. *Nat. Commun.* **10**, 4568 (2019).
